# Mining bioactive components in agricultural crop and food production residue for sustainable solutions: In silico screening for skin anti‐ageing properties

**DOI:** 10.1111/ics.13059

**Published:** 2025-04-15

**Authors:** Senka Vidović, Milan Ilić, Jelena Nakomčić, Nataša Nastić, Jelena Kvrgić, Xuanpeng Song, Dimitar Jakimov, Aleksandra Jovanović Galović, Nataša Lješković Jovanović, Mire Zloh

**Affiliations:** ^1^ Faculty of Technology Novi Sad University of Novi Sad Novi Sad Serbia; ^2^ Faculty of Pharmacy Novi Sad, University of Business Academy in Novi Sad Novi Sad Serbia; ^3^ UCL School of Pharmacy University College London London UK; ^4^ Faculty of Medicine, Oncology Institute of Vojvodina University of Novi Sad Sremska Kamenica Serbia

**Keywords:** anti‐ageing skincare, antioxidant properties, bioinformatics, collagenase inhibition, elastase inhibition, gene expression modulation, hyaluronidase inhibition, molecular docking, natural products, skin ageing

## Abstract

Possible sustainable resources of beneficial compounds for various applications are agricultural crop and food production residues (ACFPR), which are supported by considerable efforts to characterize their compositions and biological activities. This knowledge can be utilized for the rational selection of agricultural crop residue extracts and their components and possible use in the development of value‐added products, such as anti‐ageing cosmetics. The appearance of wrinkles, pigmentation, and a reduction in skin elasticity are typical signs of ageing skin that are often alleviated by natural product‐based preparations. Here, we use in silico approaches to identify natural compounds from agricultural crop and food production residues with the potential to alleviate symptoms of or reverse the skin ageing process. Target predictions combined with extensive database and literature searches were utilized to identify compounds present in ACR and proteins linked to skin ageing. The binding affinity of natural products to selected proteins using molecular docking and the respective intermolecular interaction analyses are predicted to provide an indicative measure of the compounds' potential for skin anti‐ageing activity. A number of natural compounds with the potential to interact with protein targets such as collagenase, elastase, and hyaluronidase were identified. In addition to in silico findings, cytotoxicity assays were conducted using rose hip seed extracts against Hs294T (human metastatic melanoma, ATCC HTB‐140) and MRC‐5 (normal fetal lung fibroblasts, ATCC CCL 171), demonstrating selective cytotoxicity. ELISA assays revealed that rose hip seed extracts induced a significant increase in SIRT1 levels (160% of control) and a reduction in TGF‐β levels (80% of control). These experimental results support the potential of agricultural crop residue extracts in modulating key proteins involved in skin ageing, reinforcing their viability as ingredients in anti‐ageing cosmetic formulations. An analysis of the molecular relationships and pathways that organic substances from sustainable sources can affect offers the potential for developing formulations for skin rejuvenation with possible synergistic effects by utilizing the rational design of innovative skincare products and laying the framework for more effective screening of anti‐ageing compounds for different applications.

## Graphical abstract

A workflow for identifying sustainable anti‐ageing solutions from agricultural crop and food production residues (ACFPR). A combination of in silico techniques, like database interrogations, target predictions, molecular docking, and ADME predictions can pave the way for the development of innovative, eco‐friendly anti‐ageing cosmetics.

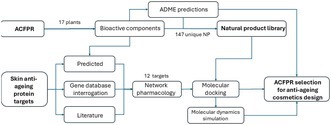

## Résumé

Les résidus de cultures agricoles et de production alimentaire (RPAA) représentent des ressources durables potentielles de composes bénéfiques pour diverses applications, soutenus par des efforts considérables visant à caractériser leur composition et leurs activités biologiques. Ces connaissances peuvent être exploitées pour une sélection rationnelle d’extraits de résidus agricoles et de leurs composants, ainsi que pour leur utilisation potentielle dans le développement de produits à valeur ajoutée, tels que les cosmétiques anti‐âges. L'apparition de rides, l'hyperpigmentation et la diminution de l'élasticité cutanée sont des signes typiques du vieillissement de la peau, souvent attenues par des préparations à base de produits naturels. Ici, nous utilisons des approches in silico pour identifier des composes naturels issus des résidus de cultures agricoles et de production alimentaire ayant le potentiel d'atténuer les symptômes du vieillissement cutané ou d'en inverser le processus. Les prédictions des cibles, combinées à des recherches approfondies dans les bases de données et la littérature, ont permis d’identifier les composes présents dans les RPAA ainsi que les protéines impliquées dans le vieillissement cutané. L'affinité de liaison des produits naturels avec ces protéines a été évaluée à l'aide de l'arrimage moléculaire et d'analyses des interactions intermoléculaires respectives, fournissant une indication du potentiel anti‐âge des composés. Plusieurs composés naturels susceptibles d’interagir avec des cibles protéiques clés, telles que la collagénase, l'élastase et l'hyaluronidase, ont été identifies. En complément des résultats in silico, des essais de cytotoxicité ont été réalisés avec des extraits de graines de cynorhodon sur les lignées cellulaires Hs294T (mélanome métastatique humain, ATCC HTB‐140) et MRC‐5 (fibroblastes pulmonaires fœtaux normaux, ATCC CCL 171), démontrant une cytotoxicité sélective. Les tests ELISA ont révélé que les extraits de graines de cynorhodon induisaient une augmentation significative des niveaux de SIRT1 (160% du contrôle) et une réduction des niveaux de TGF‐β (80% du contrôle). Ces résultats expérimentaux confirment le potentiel des extraits de résidus agricoles dans la modulation des protéines clés impliquées dans le vieillissement cutané, renforçant leur viabilité en tant qu'ingrédients pour les formulations cosmétiques anti‐âge. L'analyse des relations moléculaires et des voies affectées par les substances organiques issues de sources durables ouvre la voie au développement de formulations innovantes pour le rajeunissement de la peau, avec de possibles effets synergiques. L'utilisation d’une conception rationnelle de produits de soin pourrait ainsi poser les bases d'un dépistage plus efficace des composes anti‐âges pour diverses applications.

## INTRODUCTION

There is a significant shift towards sustainable agriculture and the development of diversified rural economies. In addition to utilizing the crop residue for energy generation [1], these materials are abundant in a diverse array of polyphenols, terpenes, alkaloids, and vitamins [2, 3]. The compounds from agricultural crop and food production residue (ACFPR) possess the capability to neutralize free radicals, absorb UV radiation, and counteract the effects of ageing‐related diseases [4]. However, the vast assortment of phytochemicals and the complex pathways associated with ageing pose challenges in identifying efficacious treatments [5]. This underscores the opportunities for potential uses of computational methods to swiftly predict the possible mechanisms of biological activities of natural products before conducting laboratory tests [6], thereby minimizing time‐intensive trial‐and‐error experiments. Such products with natural ingredients that exhibit antioxidant, anti‐inflammatory, and anti‐ageing properties are increasingly recognized as promising methods for addressing skin ageing symptoms [7].

Skin ageing is a multifaceted biological occurrence that is becoming more common in older populations, leading to significant healthcare consequences. The process is regulated by a complex interaction between genetically programmed internal mechanisms and external environmental influences, particularly ultraviolet (UV) light, which causes photo‐ageing [8]. Intrinsic ageing occurs naturally due to genetic factors, but photo‐ageing is the result of long‐term exposure to UV radiation and lifestyle choices [9]. Together, these factors result in several visible signs on the skin, such as wrinkles, dryness, looseness, and changes in skin colour, which negatively affect the overall quality of life [8, 9]. Understanding the basic foundations of skin ageing is crucial to guiding treatment therapies.

Various pathological mechanisms [10] manifest through changes in the skin's structural proteins, enzymes, and pigmentation control systems, aligning with the observable signs of ageing [11]. The structural integrity of the skin is undermined by the degradation of collagen and elastin, primarily due to the increased activity of matrix metalloproteinases. Concurrently, the skin's moisture and fullness are compromised by a decline in hyaluronic acid production, attributed to inhibited hyaluronidase activity. The repair and renewal capabilities of the skin are further impacted by the breakdown of fibronectin and laminin, which are essential for fibroblast migration and proliferation, thus impairing the skin's healing processes. Additionally, the build‐up of reactive oxygen species, exacerbated by ultraviolet radiation and inflammatory cytokines, amplifies tyrosinase activity, leading to alterations in skin pigmentation. The sirtuin family of proteins plays a pivotal role in skin ageing by influencing genomic stability, DNA repair, chromatin structure modification, and stress response through their deacetylase functions [12, 13, 14, 15]. The ageing of the skin, therefore, results from a series of synchronized molecular events that collectively weaken the skin's structure and function. Investigating these molecular pathways offers insights into potential therapeutic targets that could offer mitigation of the effects of skin ageing [16].

Virtual screening could serve as a framework for the systematic evaluation of ACFPR influence on skin ageing, facilitated by bioinformatics and computational modelling. Molecular docking predicts the potential strength and mechanism of interaction between targets and ligands, offering a theoretical estimation of their binding affinity and mode. This method provides preliminary insights that are subject to experimental validation to ascertain their true activity [17], including an understanding of the skin antiaging activities of natural products [18].

The identification of relevant target proteins integral to the skin ageing process is an essential and integral step [19] in the possible utilization of agricultural crop residues. These residues, typically overlooked, may harbour compounds with anti‐ageing capabilities. This study aims to aid the development of novel anti‐ageing treatments that integrate agricultural sustainability using in silico approaches. The objectives are to assess the compounds' potential to mitigate skin ageing by interacting with protein targets and to explore potential synergistic actions of compounds in different extracts of common agricultural crop residue from the Vojvodina province and Serbia. This computational prioritization is poised to significantly enhance the efficiency of subsequent in vitro development of promising treatments. Our effort is to pave the way for new anti‐ageing therapies by capitalizing on the untapped potential of agricultural crop residues, thus fostering a sustainable future in agriculture, food production, and skincare.

## MATERIALS AND METHODS

### 3D structural library of natural products from agricultural crop residues

A set of natural product molecules present in agricultural crop and food production residues, including leaves, branches, peels, and seeds was collated from the literature using Google Scholar, PubChem, and PubMed resources. These sustainable sources available in Vojvodina province and the rest of Serbia include apples (leaves, pomace, skin), beetroots (leaves, root), blueberries (fruit, leaves), carrots (leaves, peel, pomace), cherries (fruit), chokeberries (leaves, pomace, stems), citrus fruit (peel), cranberries (fruit, juice, leaves), garlic, grapevines (green pruning, leaves, pomace, seed, skin, stalks), onions (apical trimming, leaves, outer dry layer, skin), peppers (leaves, seed), plums (leaves, seed, skin), rosehip (herbal dust) and tomatoes (leaves, seed, skin). The literature search resulted in a collection of entries with duplicate molecules that are coming from different sources. The Simplified Molecular Input Line Entry System (SMILES) strings for a set of 172 unique entries were retrieved from PubChem [20] and imported into DataWarrior v06.02.01 [21] and a range of physicochemical and toxicity properties were predicted.

The SMILES strings were used to generate the molecules' three‐dimensional (3D) conformations using Open Babel, an extensively used open‐source tool for converting and handling chemical data files [22]. The generated models included hydrogen atoms to reflect the ionizable groups' charge state, for a pH of 7.4 and the structures were minimized to the most probable local minimum energy state using molecular mechanics and MMFF94 force field. The final 3D conformations were saved as Mol2 files.

### Identification of protein targets

The SMILES strings were utilized for the prediction of the putative drug targets for all identified natural products using the SwissTargetPrediction (http://www.swisstargetprediction.ch/) [23] and Epigenetic target profiler v1.0 (http://www.epigenetictargetprofiler.com/) [24] web servers. The ‘*Homo sapiens*’ option was selected for the SwissTargetPrediction runs, and only targets with a probability greater than 0 were recorded. The predicted epigenetic targets with a consensus prediction by two methods (Morgan SVM and RDK SVM) were used for further analysis.

Drug targets relevant to the skin ageing process were searched using the Human Gene Database server (https://www.genecards.org/) [25] using keywords ‘+skin +antiageing’ Additionally, relevant protein targets known to be used in computational studies were identified using a literature review conducted with Google Scholar. Specific keywords such as ‘Docking’, ‘Anti‐ageing’, ‘Skin’, and ‘Protein’ were used to ensure a targeted and relevant collection of scholarly articles. This process resulted in potential protein targets within the skin that play a critical role in ageing and related dermatological conditions.

The intersection of the predicted targets for the compounds predicted for all selected molecules and skin ageing targets was obtained using Venny 2.1 (https://bioinfogp.cnb.csic.es/tools/venny/) [26] and the network of intersecting targets was constructed using Cytoscape 3.10.2 [27].

### Protein structure preparation

A subset of common targets with available 3D structural data in the Protein Data Bank (PDB) [28] was selected for further molecular docking studies. The proteins selected from the literature review for their relevance to skin ageing and potential as therapeutic targets include collagenase (1CGL, 2TCL), elastase (1ELB), fibronectin (1FNF), hyaluronidase (2PE4), laminin (5XAU), matrix metalloproteinase‐2 (8H78), transforming growth factor‐beta type I receptor (1VJY), tumour necrosis factor‐alpha (2AZ5), tyrosinase (7RK7 and AlphaFold model P14679‐F1), sirtuin 1 (4I5I), and sirtuin 6 (3 K35).

These selected structures were prepared for molecular docking using VegaZZ 3.2.3.28 [29]. The protein models were processed to remove water molecules, add missing hydrogen atoms, and assign appropriate charge states to the amino acid residues. The coordinates of the centre of the ligands were recorded (Table S1) and ligands were removed from the binding sites. The exception was the centre of the box for the AlphaFold model of tyrosinase, which had no ligand present, and the protein centroid was used for blind docking.

### Computational docking and analysis

Molecular docking was conducted using Autodock Vina [30] with the Vegas ZZ module as a graphical user interface for the Vina software. The potential binding affinities of agricultural crop residues with the prepared skin protein targets were evaluated. A script was executed to conduct virtual screening with the docking box size set to accommodate the largest of the ligands (24 Å x 24 Å x 24 Å) and the docking box's centres mainly aligned with the active site's geometric centre of the ligand or, lacking such data, the protein structure's centroid as predicted by a Vina script (Table S1). The search exhaustiveness, determining docking process thoroughness, was set at 50, optimizing computational time and stable pose identification likelihood. Generally, greater exhaustiveness enhances optimal conformation discovery. Five binding modes (poses) were recorded, offering a spectrum of interactions for analysis and promising candidate selection for skin condition therapeutics. These parameters ensure the docking study's robustness and validity, which was also confirmed by redocking a ligand from the crystal structure and confirming that the deviation of its docked pose from the X‐ray conformation was lower than 2 Å.

The crop residues with the highest scores were subjected to detailed visualization and analysis using Biovia Discovery Studio Visualizer v21.1.0.20298 [31], which was used for the generation of 2D and 3D protein –ligand plots.

### Cell lines

The study utilized two cell lines: Hs294T (ATCC HTB‐140, a human metastatic melanoma cell line) and MRC‐5 (ATCC CCL 171, normal fetal lung fibroblasts). These cells were cultured in Dulbecco's modified Eagle's medium (DMEM) containing 4.5% glucose, supplemented with 10% fetal calf serum (FTS, Sigma‐Aldrich, St. Louis, MO, USA), as well as antibiotics and antimycotics (Sigma‐Aldrich, St. Louis, MO, USA). Culturing was performed in Costar 25 cm^2^ flasks at 37°C, with an atmosphere maintaining 100% humidity and 5% CO₂ (Heraeus, Hanau, Germany). All assays were conducted using viable cells in the exponential growth phase.

### MTT assay

Cell growth inhibition was assessed using the tetrazolium colorimetric MTT assay (Sigma‐Aldrich, St. Louis, MO, USA) [32, 33]. This assay relies on the reduction of the tetrazolium salt MTT (3‐(4,5‐dimethylthiazol‐2‐yl)‐2,5‐diphenyl tetrazolium bromide) to formazan by mitochondrial dehydrogenases in living cells. Exponentially growing cells were harvested, counted using trypan blue, and seeded into 96‐well microtiter plates (Costar) at a density of 10,000 cells per well to ensure logarithmic growth throughout the assay. Each well contained 90 μL of medium, and cells were pre‐incubated at 37°C for 24 h to stabilize before adding test substances. Substances were added at 10 times the required final concentration (10 μL per well), except in control wells, and incubated for 24 and 48 h. Control wells contained cells without test substances. Three hours before the end of the incubation, 10 μL of MTT solution (5 mg/mL, filtered to sterilize and remove insoluble residues) was added to each well. Post‐incubation, 100 μL of acid‐isopropanol (0.04 N HCl in isopropanol) was added to dissolve the formazan crystals. Plates were then read on a spectrophotometer (Multi‐scan MCC340; Labsystems) at 540/690 nm. Blank wells containing medium and MTT without cells were used for reference. Growth inhibition was calculated as a percentage of the control using the formula: (1 − A_test/A_control) × 100. The potency of the test substances was expressed as the IC50 value (50% inhibitory concentration). Each experiment was performed in triplicate, and results were reported as mean values.

### SIRT1(Sirtuin1) and TGF beta 1 ELISA tests

Cell extracts of the Hs294T cells (for sirtuin 1 assay) and cell culture supernatants (for transforming growth factor beta 1 assay) were prepared according to the manufacturer's instructions. The human sirtuin1 and TGF beta 1 were analysed with ELISA kits (Abcam, ab171573 & ab100647, respectively) according to the manufacturer's instructions.

## RESULTS

### Library of natural products from ACFPR

The literature search of the composition of ACFPRs has yielded a set of 526 entries (Data S2: Worksheet S1) that had some molecules occurring multiple times for different plants. The compilation was completed in 2022 and it is not comprehensive due to the different limitations that could not be resolved despite best efforts, including a limited number of studies available for different ACFP types. Additionally, there is very limited quantitative data on the composition of these compounds, so that data was not collated at this stage.

The most abundant information is available for tomato (19 components in leaves, 56 in seed, 47 in skin, 122 in total), followed by information about the composition of components of plum residue (54 components in leaves, 15 in seed, 27 in skin, 96 in total). The other well‐studied compounds are blueberry (72 components in total) and cranberry (58 components in total); however, some information is available from either juice or fruit, and therefore not strictly speaking from crop or production residue.

The removal of duplicates resulted in a list of 174 unique components for which structures and SMILES strings are available (Data S2: Worksheet S2). Predicted physchem properties by the DataWarrior algorithm, as expected, indicate that the compounds have variable lipophilicity and solubility, with a few having oral drug‐like properties (39 compounds). On the contrary, less than 15% of compounds have a high probability of acting as mutagenic agents, while the percentages of compounds acting as irritants or tumorigenic agents are even lower (less than 10% on average).

### Skin ageing target identification

The SMILES library of ACFPR compounds was used to predict their drug targets by interrogating the SwissTargetPrediction server. The total number of protein targets with a probability of compounds being able to interact with them was 701 proteins, but only 160 proteins are considered as the top six hits for each molecule (Data S2: Worksheet S3). The top 10 drug targets with their frequency of being involved in the interactions are shown in Table 1. The AKR1B1 aldo‐keto reductase family 1 member B, a protein responsible for catalysing the reduction of aldehydes, shows the highest promiscuity by being a top hit for 36 components, which indicates that some of the extracts can affect pathological processes in diseases such as diabetic retinopathy, cardiovascular disorders, and possibly cancer. The cluster of differentiation 38 (CD38) target is known to be linked with an impaired immune system, and its inhibitors could be used in the treatment of asthma.

**TABLE 1:** Top ten predicted targets for components of ACPFR.

Drug targets			Epigenetic targets		
Protein	Frequency	Frequency as top‐hit	Gene	Frequency	Frequency as top‐hit
AKR1B1	83	36	APEX1	159	145
CD38	52	12	ATM	154	10
CA2	98	10	KDM4E	120	6
PPARA	22	9	CDK1	119	0
NOX4	58	7	BRD2	92	1
CYP19A1	27	6	AURKB	83	1
PTGS2	54	6	HDAC1	77	1
PPARG	10	6	BRD4	76	0
MMP2	32	5	PARP1	62	2
NMUR2	52	5	PRKCD	51	0

*Note*: Drug targets were predicted using the SwissTargetPrediction server, and epigenetic targets were predicted using the Epigenetic Target Profiler. Predictions used SMILES strings.

The number of predicted epigenetic proteins that could potentially be targets for ACFPR components is lower; based on the consensus of two methods, 47 proteins could be interacting with the components (Data S2: Worksheet S4). Although the implications of the APEX1‐small molecule interactions are not fully understood on a whole‐body level, there are some potential benefits of targeting epigenetic regulators. Targeting ATM could have beneficial effects on DNA repair with positive effects in cancer patients and neurodegenerative disorders, but could also disrupt the balance between activation and repression of genes.

The above results confirm that ACFPR has the potential to affect biological processes and the range of such processes is quite varied as indicated via a limited number of examples. As a case study for the use of ACFPR extracts, their potential application in minimizing the effects of skin ageing is explored. Therefore, relevant protein targets were searched for in the Human Gene Database. A total of 139 entries were found to contain both skin and anti‐ageing keywords Data S2: Worksheet S5, with 91 classified as protein‐coding.

**FIGURE 1:**
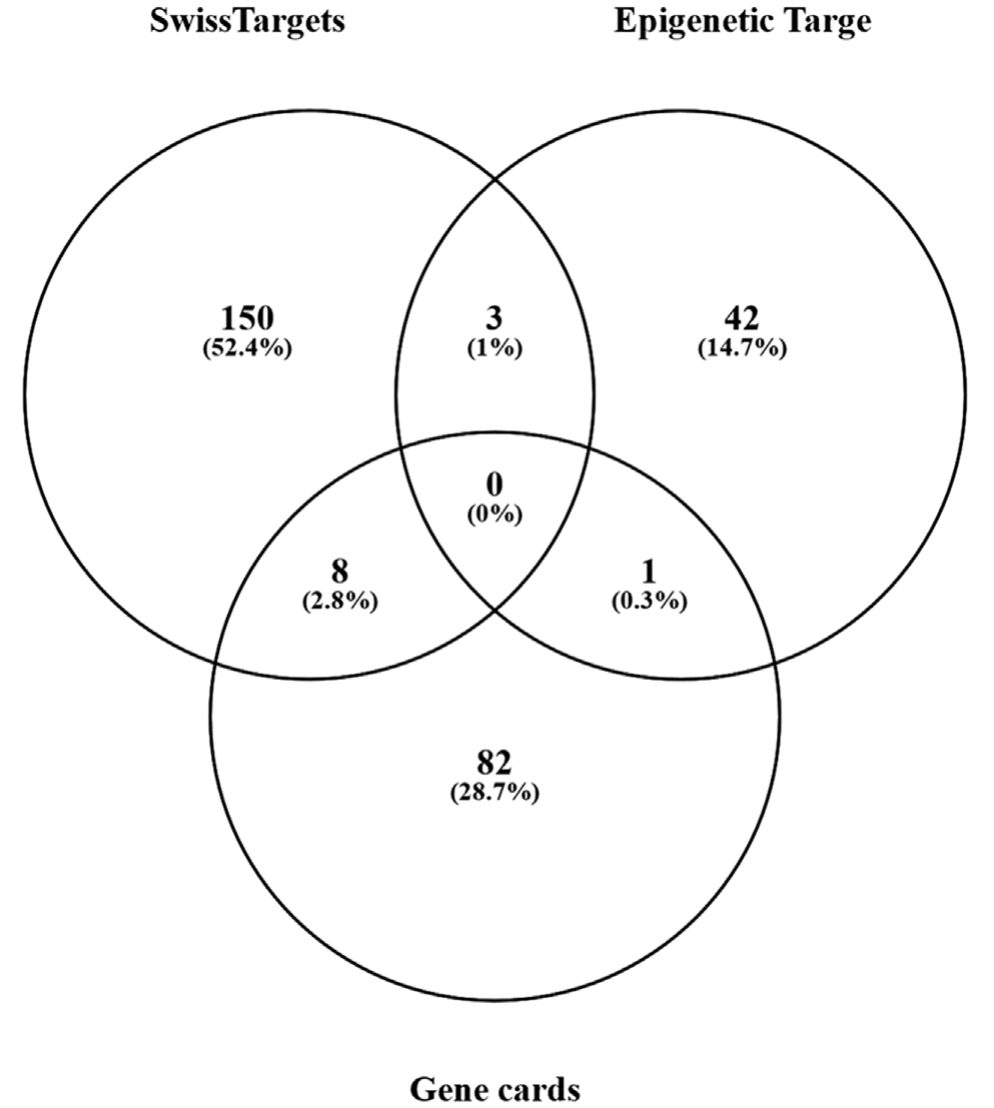
Overlapping target proteins found in the Human Gene Database (protein‐encoding genes that are related to skin anti‐ageing) and drug targets of ACFPR compounds predicted by SwissTargetPrediction and Epigenetic Target Profiler.

These protein‐encoding genes were intersected with predicted targets using the Venny tool and it was found that 8 targets are common in the Gene Database and library predicted by the SwissTargetPrediction tool (Figure 1). These intersecting drug targets are different to those found in the previous studies related to skin anti‐ageing, however, MMP1 and SIRT1 are found in the list of the proteins that are not intersecting (Table 2).

**TABLE 2:** List of overlapping predicted protein targets and those found in the literature that are used for docking studies.

STP ∩ GC	ETP ∩ GC	STP ∩ ETP	Literature
TNF	CDK5	HDAC1	MMP1
TERT		KDM4E	FN1
MMP2		TOP2A	HYAL1
MMP9			SIRT1
BCL2			SIRT6
PPARA			LAMA5
GSK3B			TYR
NQO2			CELA1

*Note*: STP, GC, ETP, and ∩ depict SwissTargetPrediction, Gene Card, Epigenetic Target Profiler, and the intersect symbol, respectively.

### Molecular docking for anti‐skin ageing activity evaluation

The natural compounds known to be in ACFPR extract were docked against a set of skin anti‐ageing protein targets found by a combination of approaches. The receptors selected for this investigation were crucial skin proteins, encompassing particular enzymes acknowledged for their substantial contributions to skin well‐being and ailments. The target proteins included collagenase, hyaluronidase, laminin, fibronectin, sirtuin, and tyrosinase. Each of these proteins plays a crucial role in skin physiology and the ageing process, serving as important markers. Their individual PDB codes were utilized to identify and retrieve their structural data for the docking simulations. The coordinates of the active site for each protein receptor were established and setup as grid boxes using Autodock Vina to conduct the docking. This ensured that the predicted interactions took place at the biologically relevant sites of the selected proteins.

The results of docking (Data S2: Worksheet S6) provide insight into predicted individual binding scores as well as average values. The heat map in S2: Worksheet S6 and average binding scores per protein clearly indicate that natural products favourably bind to most proteins (average binding scores range from −6.3 to −8.2 kcal/mol). However, most favourable binding scores achieved even better docking scores, namely Sirtuin 1 (−11.5 kcal/mol), laminin (−11 kcal/mol), and Sirtuin 6 (−10.7).

**FIGURE 2:**
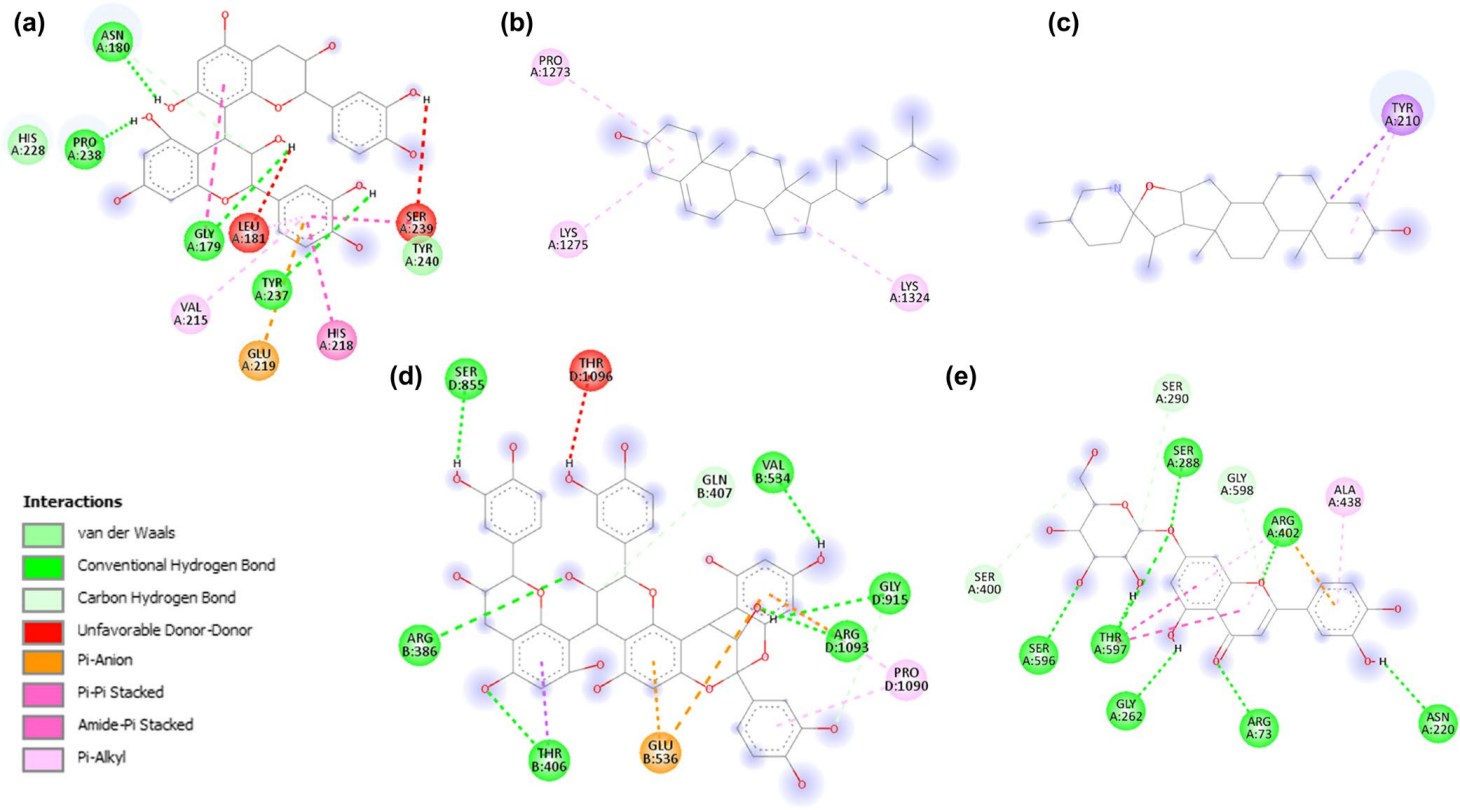
2D interaction diagrams demonstrating the molecular docking analysis of selected skin protein targets with their respective ligands exhibiting the highest binding affinities. Each panel illustrates the intricate binding site interactions, highlighting the ligand's potential inhibitory mechanisms: (a) depicts the interaction between 1CGL and procyanidin B1; (b) shows campesterol docked with 1FNF; (c) visualises the binding of tomatidine to 2PE4; (d) presents cinnamtannin B1 engaged with 3K35; (e) illustrates the interaction of luteolin‐7‐O‐glucopyranoside (cynaroside) with 5XAU. These diagrams are derived from the binding energies recorded in (Table 2), providing a visual insight into the molecular interactions that underpin the docking results.

An example of interactions between selected compounds with various biological targets, such as sirtuin 6, tyrosinase, collagenase, fibronectin, hyaluronidase, laminin, and their average binding scores per target is shown in Table 3. with their protein–ligand plots in Figure 2.

**TABLE 3:** The docking scores (kcal/mol) of selected ligand‐receptor complexes were predicted using AutoDock Vina.

Ligands	1CGL	1FNF	2PE4	3K35	5XAU	7RK7
Tomatine	−9.4	−7.9	−8.8	−10.3	−9.3	−10.4
Cinnamtannin B1	−9.4	−7.4	−8.4	−10.7	−9.1	−10.3
luteolin‐7‐O‐glucopyranoside (Cynaroside)	−8.9	−7.2	−7.9	−8.8	−9.6	−10.6
Campesterol	−7.8	−8.3	−8.6	−10.3	−7.6	−9.4
Procyanidin B1	−8.8	−7.7	−7.5	−9.1	−10	−9.6

These ligands, extracted from agricultural crop residues, include tomatine from tomato leaves, cinnamtannin B1 from cranberry leaves, luteolin‐7‐O‐glucopyranoside (cynaroside) from pepper leaves, campesterol from cranberry leaves, and procyanidin B1 from plum skin and apple peel. The understanding of their binding affinities is crucial for the progression of research into bioactive compounds.

The ligand tomatine exhibits remarkable binding affinities, ranking among the highest observed in our docking output. Tomatine demonstrated the highest affinity for the 7RK7 protein target, with a binding strength of −10.4 kcal/mol, as shown in Table 3. This emphasizes its potential to act as a potent inhibitor. The interaction between the 3 K35 protein and this compound was particularly noteworthy, exhibiting a binding affinity of −10.3 kcal/mol. The consistent high binding affinities of tomatine for various protein targets, such as fibroblast collagenase (1CGL: −9.4 kcal/mol), fibronectin (1FNF: −7.9 kcal/mol), hyaluronidase‐1 (2PE4: −8.8 kcal/mol), and laminin‐511 (5XAU: −9.3 kcal/mol), highlight its potential to act as a versatile inhibitor.

Cinnamtannin B1 demonstrated high binding affinities, particularly with the sirtuin‐6 (3 K35: −10.7 kcal/mol) and the 7RK7 protein (−10.3 kcal/mol), as shown in Table 3. The compound exhibited strong inhibitory potential against various proteins, as indicated by its affinities of −9.4 kcal/mol for 1CGL, −7.4 kcal/mol for 1FNF, −8.4 kcal/mol for 2PE4, and − 9.1 kcal/mol for 5XAU.

Cynaroside, also known as luteolin‐7‐O‐glucopyranoside, was found to be an exceptionally effective ligand. It displayed its strongest binding affinity of −10.6 kcal/mol towards the 7RK7 protein target, as indicated in Table 3. The compound demonstrated uniform and substantial binding affinities for all tested targets: −8.9 kcal/mol for 1cgl, −7.2 kcal/mol for 1FNF, −7.9 kcal/mol for 2PE4, −8.8 kcal/mol for 3 K35, and − 9.6 kcal/mol for 5XAU. This pattern indicates the potential for targeted inhibition that could be utilized in therapeutic settings.

Campesterol exhibited significant interactions, particularly with the 3 K35 receptor, demonstrating a binding affinity of −10.3 kcal/mol, as stated in Table 3. Although its binding affinities were slightly lower compared to the strongest binders, it still displayed noteworthy interactions. The binding affinities of campesterol for different targets were as follows: −7.8 kcal/mol for 1CGL, −8.3 kcal/mol for 1FNF, −8.6 kcal/mol for 2PE4, −7.6 kcal/mol for 5XAU, and −9.4 kcal/mol for 7RK7. These values suggest that campesterol has the potential to act as a moderate inhibitor.

Procyanidin B1, which is included in our list of top ligands, exhibited significant binding affinities. Specifically, it displayed binding affinities of −8.8 kcal/mol for 1CGL, −7.7 kcal/mol for 1FNF, −7.5 kcal/mol for 2PE4, −9.1 kcal/mol for 3 K35, −10 kcal/mol for 5XAU, and − 9.6 kcal/mol for 7RK7, as shown in Table 3. These values reinforce the ligand's interaction profile with the protein targets, affirming its potential as a therapeutic molecule.

Following the previous steps of molecular docking, we have utilized the Biovia Discovery Studio Visualizer to generate a comprehensive visualization of the protein binding pockets. This diagram (Figure 2) is a critical output of our computational analysis, revealing the intricate interactions between natural compounds and proteins implicated in the skin ageing process. By meticulously mapping these interactions, we can better understand the molecular underpinnings of ageing and identify natural compounds that may serve as effective inhibitors. The visualization provides a detailed overview of the binding affinities and interaction patterns, which are essential for the subsequent stages of our research, including empirical testing and potential therapeutic applications.

The molecular interaction map offers a granular perspective on the ligand‐protein interactions. Figure 2a captures the multifaceted interaction of procyanidin B1 with protein 1CGL, where a benzene ring engages in π‐alkyl with V215, π‐π stacked with H218, and additional interactions with E219. Figure 2b displays campesterol docked with 1FNF, forming alkyl interactions with P1273, K1275, and K1324. Figure 2c visualizes tomatidine binding to 2PE4, with van der Waals forces from three ligands and Y210 engaging in π‐sigma and π‐alkyl interactions with a hexene ring. Figure 2d shows cinnamtannin B1 engaged with 3 K35, where E536 forms π‐anion interactions with two benzene rings, R1093 engages in π‐anion and conventional hydrogen bonding with a benzene ring, and T406 establishes a π‐sigma bond and a conventional hydrogen bond with an oxygen atom on the same ring. Lastly, Figure 2e illustrates interaction of luteolin‐7‐O‐glucopyranoside (cynaroside) with 5XAU, where Y547 forms a π‐π stacked interaction with a benzene ring, which also interacts with A69 and K68 through π‐alkyl interactions. Additionally, K68 forms an alkyl interaction with another ring featuring a single double bond, underscoring the complexity of these molecular interactions.

The computational analysis depicted in Figure 2a through 2e offers a hypothetical framework for understanding how natural compounds might interact with proteins linked to skin ageing. The array of interactions, including pi‐alkyl, pi –pi stacked, and conventional hydrogen bonds, implies the possibility of these compounds forming complexes with the proteins, which could, in theory, influence the proteins' functions and potentially modulate the ageing process. The diversity of interaction types observed may reflect a level of specificity that could be beneficial in the design of compounds targeting the ageing pathway. These computational predictions provide a foundation for further empirical research to validate the potential of these compounds as anti‐ageing agents [24].

### Experimental validation of protein extract interactions

**FIGURE 3:**
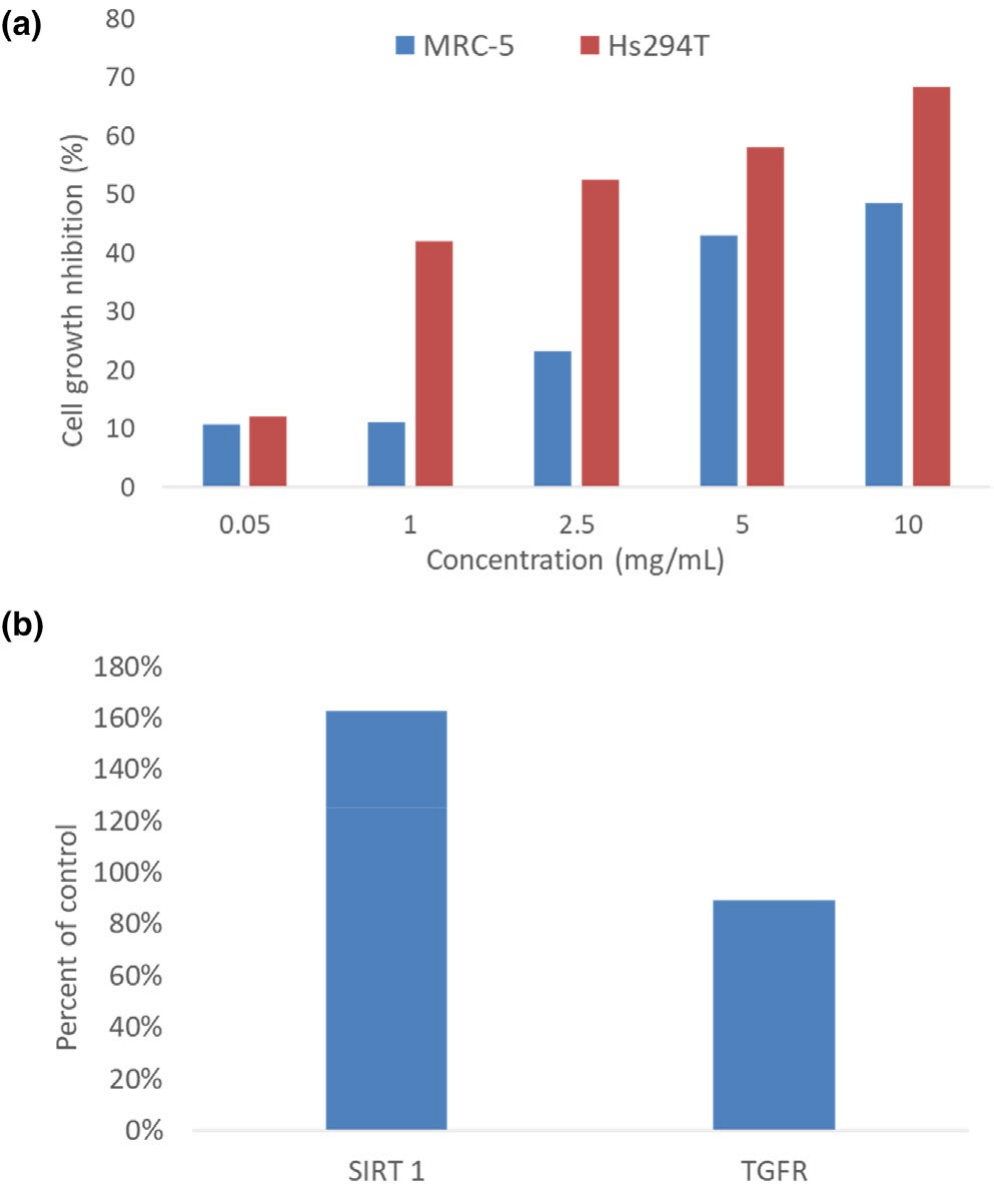
In vitro assessment of the rose hip extract: (a) cell growth inhibition determined by MTT test on MRC‐5 and HSl cell lines after exposure to rosehip extract in concentrations from 50 μg/mL to 125 mg/mL; (b) ELISA results showing the effect of rosehip dust extract on the levels of SIRT1 and TGF‐beta in Hs294T cell lines compared to control.

The extract obtained from the herbal dust waste during rose hip filter tea production [34] was evaluated for its cytotoxic activity and expression levels of two proteins relevant for skin ageing, Sirtuin 1 (SIRT1) and, transforming growth factor‐beta type I (TGF‐beta). Cytotoxicity assays were performed using rose hip seed extracts on two cell lines: Hs294T (human metastatic melanoma, ATCC HTB‐140) and MRC‐5 (normal fetal lung fibroblasts, ATCC CCL 171). The extracts exhibited selective cytotoxicity, showing greater activity against Hs294T melanoma cells compared to MRC‐5 fibroblasts, especially for 1 mg/mL and 2.5 mg/mL concentrations (Figure 3a). Such selective cytotoxicity suggests a potential therapeutic window for targeting melanoma cells with acceptable activity against normal cells.

ELISA assays were conducted to quantify the levels of SIRT1 and TGF‐beta in treated cell lines. Cells treated with rose hip seed extracts exhibited a significant increase in SIRT1 levels, reaching 160% of the control, and a reduction in TGF‐beta levels to 80% of the control. This indicates that the extracts may enhance SIRT1 expression, which is linked to anti‐ageing effects, while simultaneously reducing TGF‐beta levels, potentially mitigating fibrosis and inflammation.

The compounds known to be present in the extract, including campesterol, sitosterol, and tocopherols [34] were found in a library created in this study. These molecules can form favourable interactions with key proteins associated with skin ageing, including SIRT1 (PDB ID: 4I5I). The range of average docking scores is between −7.6 and −8.2 kcal/mol and maximum values up to −11.4 kcal/mol (Data S2: Worksheet S4). The molecular docking studies demonstrated significant binding affinities, indicated strong interactions, suggesting that the compounds in the extracts can effectively bind and potentially modulate the activity of these targets, which is indirectly confirmed by our in vitro experiments. This is also supported by the known use of rose hip extracts in various dermatological formulations [35] in concentrations that are similar to those found to be optimal in this study [36].

### Case study – Plum seed extract

The variation in binding affinities of components of extracts illustrates the complex network of interactions between small molecules and protein targets, underscoring the importance of thorough screening in identifying lead compounds with outstanding specificity and efficacy. However, the selected examples belong to different ACFRP extracts, and therefore it is important to identify a single extract that could potentially be used in cosmetic formulations. The average docking scores per compound (Data S2: Worksheet S6. Column O) could indicate the affinity of a molecule to various targets. Some of the 28 molecules with average binding scores within the top 20% of the whole set can be found in most extracts; however, the highest number of best binders is found in blueberries (10), plums (9), and cranberries (7). As data for blueberry and cranberry material includes fruit and juice, particular attention will be put on plum residue extract. The eight compounds are found in the skin, and two are found in seed (epicatechin and rutin). However, the main plum residue that contains skin is obtained after distillation in the production of plum brandy. The available data does not specify if the skin composition is obtained before or after distillation; therefore, focus was moved to the possible use of plum seed extract.

An additional literature search unravelled the possible presence of additional compounds, for which only 66 out of 548 proteins predicted using the SwissTargetPrediction server were in the top six hits. Out of these, 45 targets were unique and six were in common with the results of Gene Cards (Data S2: worksheet S7 and Figure S1). However, for a new docking study, a subset of proteins was selected, and the docking scores indicate that all molecules can form favourable interactions with targets relevant to skin anti‐ageing (Table 4).

**TABLE 4:** The binding scores of selected plum seed extract complexed with collagenase (1CGL), elastase (1ELB), tumour necrosis factor‐alpha (2AZ5) and sirtuin 1 (4I5I).

Name	Docking score (kcal/mol)			
	1CGL	1ELB	2AZ5	4I5I
Epicatechin	−8	−7.2	−8.6	−10.7
(E)‐3,3′‐dimethoxy‐4,4′‐dihydroxy stilbene	−6.5	−6.5	−6.8	−8.7
5‐hydroxymethylfurfural	−5.5	−4.5	−4.5	−5.6
Amygdalin	−8	−7.5	−7.8	−10.3
Coniferyl aldehyde	−6.1	−5.2	−5.9	−7.1
Coumaric acid	−7	−5.5	−5.9	−7.1
Dihydrodehydrodiconiferyl alcohol	−7.1	−7.1	−7.4	−8.7
Epigallocatechin	−7.3	−6.7	−7.5	−9.4
Ferulic acid	−6.6	−5.5	−6.1	−7.4
Ficusal	−6.2	−6.7	−7.6	−9.8
Gallic acid	−6.6	−5.2	−5.8	−7
Quercetin‐3‐O‐rutinoside (rutin)	−8.3	−8	−8.3	−8
Scopoletin	−7.6	−5.3	−5.9	−7.3
Syringic acid	−5.4	−5.3	−5.4	−6.5

*Note*: The docking scores were predicted using AutoDock Vina.

The best binding scores were obtained for docking poses of compounds in the sirtuin 1 binding site, which is in line with observed trends within the whole set. Although the predicted docking scores are lower compared to some of the observed values in Data S2: Worksheet S6, these values indicate at least comparable if not better potential to interact with skin anti‐ageing proteins to those reported elsewhere [18]. Therefore, further in vitro exploration of the plum seed extract future use in cosmetic preparations is warranted.

## DISCUSSION

This study has offered valuable insights into the utilization of compounds derived from value‐added agricultural products in the field of skin anti‐ageing. Compounds such as tomatine, cinnamtannin B1, luteolin‐7‐O‐glucopyranoside (cynaroside), campesterol, and procyanidin B1 have been identified due to their significant interactions with skin proteins, which are essential for their potential involvement in anti‐ageing treatments. The inhibition of proteins such as sirtuin, tyrosinase, collagenase, fibronectin, hyaluronidase, and laminin has been correlated with positive effects on skin anti‐ageing in previous studies. Their inhibition correlates with improved skin resilience, even tone, firmness, repair, hydration, and texture respectively [37].

The utilization of natural products in cosmetic formulations has been a cornerstone of the industry, with a history of incorporating botanical extracts and compounds for their therapeutic properties. In the realm of anti‐ageing, these natural constituents have been selected for their ability to synergise with the skin's biological mechanisms [7]. For instance, the employment of flavonoids, such as luteolin‐7‐O‐glucopyranoside, has been prevalent due to their potent antioxidant activity, which combats free radical damage and promotes collagen synthesis [38]. Similarly, phytosterols like campesterol have been integrated into creams and serums for their resemblance to cholesterol, a key component of the skin's lipid barrier, thus enhancing moisture retention and structural integrity [39].

Additionally, the whole extract of plum seed also has a significant potential to interact with these skin protein targets. It has already been shown that epicatechin and epigallocatechin can be used in skin protection from UV radiation [40], while it was found that 5‐hydroxymethylfurfural exerted anti‐inflammatory activity in skin disorders [41] and that rutin has significant biological effects on skin ageing [42]. Other compounds from the plum seed extracts are also known for their use in anti‐ageing formulations as reported in many studies [43, 44], but this does not present a comprehensive review of all uses. These established results form the foundation for the validation of this study, which explores the anti‐ageing potential of compounds found in agricultural crop residues without prior bias on the compound selection.

The transition from traditional, holistic applications to targeted, mechanistic utilization reflects a paradigm shift in cosmetic science [45]. This shift is underscored by scientific validation of these compounds' efficacies, such as through molecular docking studies, which ascertain their interactions with skin proteins pivotal in the ageing process. The selection of crop residues in this study underscores a commitment to environmental sustainability, as these by‐products represent a significant source of underutilized biomass [46]. The utilization of such residues aligns with the principles of circular economy, where waste materials are repurposed to create value‐added products, thereby reducing the environmental footprint of agriculture [47]. In the context of cosmetic applications, the incorporation of compounds derived from agricultural by‐products not only mitigates the disposal issues associated with these residues but also contributes to the reduction of greenhouse gas emissions associated with their decomposition or incineration. Furthermore, this approach promotes the conservation of biodiversity by reducing the demand for virgin raw materials and minimizing the impact on natural ecosystems. By harnessing the potential of agricultural by‐products, this study contributes to the development of sustainable and eco‐friendly cosmetic solutions, reflecting a paradigm shift towards greener and more responsible science [48].

The molecular docking techniques combined with literature and databases interrogation aided the evaluation of binding affinities of plant‐derived ligands, specifically those obtained from value‐added agricultural products, to protein targets involved in the skin ageing process. This computational approach is based on tested models, offering a fast and cost‐effective alternative to traditional experimental methods. Expanding on the groundwork laid by previous studies, we followed established procedures to guarantee the accuracy of our computer‐generated forecasts [49]. The choice of ligands derived from agricultural by‐products not only demonstrates an innovative approach to integrating sustainability into scientific research but also aligns with the increasing consumer demand for natural ingredients and sustainability in skincare.

The computational outputs suggest that the simultaneous delivery of several molecules on the skin may offer anti‐ageing benefits. By potentially targeting multiple pathways, these combinations could work together to combat the signs of ageing more effectively than single agents. For example, the theoretical application of various molecules could offer a range of anti‐ageing effects, such as mitigating oxidative damage, promoting skin hydration, and supporting collagen integrity [50]. These effects, while not directly observed in the study, represent possible outcomes of a synergistic approach to skincare based on computational predictions. This multifaceted approach has the potential to improve the appearance of the skin by reducing wrinkles and increasing firmness, as well as possibly enhancing the skin's overall health and resilience against environmental stressors [51]. The strategic use of nanocarriers may further optimize the delivery and efficacy of these molecules from crop residues, potentially ensuring that they reach the deeper layers of the skin where they can exert their beneficial effects [52]. Ultimately, the integration of these advanced delivery systems with a synergistic blend of anti‐ageing molecules holds promise for the development of more effective skincare treatments, although further research is needed to confirm these effects.

Nevertheless, the intricate nature of biological systems poses difficulties that our computational techniques may not comprehensively address. An example of this is the limited capability of molecular docking to consider the dynamic alterations in protein structure, the effects of the cellular surroundings, and the possible consequences of solvent interactions. This may result in an oversimplified representation of the biological reality. These factors may lead to differences between the anticipated and actual binding affinities, thereby serving as a constraint to the study [53]. Although molecular dynamics simulations can be particularly useful in general drug discovery and hit confirmation, the situation is far more complex for the behaviour of these natural products in cosmetic formulations and their interactions with the proteins in the skin. Pilot molecular dynamics simulation in a system with explicit water shows that protein–ligand interactions may not be stable over 100 ns trajectories (Figure S2). However, as that is not a realistic representation of the system studied, the implicit solvent molecular dynamics simulations could be used as a screening method for testing the stability of the formed complexes (Figures S3 and S4). This could be further extended into molecular dynamics systems of more complex systems that explicitly include all components of the extract, formulation, and skin environment. Furthermore, the lack of experimental confirmation of the anticipated interactions using in vitro or in vivo testing is a significant limitation that prevents making a definitive statement about the biological activity of the ligands under consideration.

The outcomes obtained from our molecular docking investigations are inherently relative, as opposed to being absolute, because of the characteristics of the docking algorithms and the scoring functions employed to assess binding affinities [53]. The purpose of these scoring functions is to prioritize ligands based on their anticipated capacity to interact with a target protein, offering a comparative evaluation of binding potential rather than an absolute quantitative assessment. The scoring systems consider multiple molecular interactions, including hydrogen bonding, hydrophobic effects, and electrostatic complementarity, to generate a score that predicts the binding affinity between a ligand and a protein [54]. Nevertheless, this score is an abstract concept, obtained from a simplified representation of intricate biological interactions. Although these models can provide valuable information and indicate patterns or possible results, they do not produce precise quantitative data that can only be obtained through experimental verification. Therefore, it is important to interpret the results as indications of potential binding strength rather than as exact measurements of actual binding affinity. The comparative analysis is essential for determining the priority of compounds for further investigation. However, it is necessary to conduct subsequent empirical validation to confirm the biological significance of the predicted interactions [55].

Future research should focus on closing the disparity between computational predictions and empirical evidence. Performing in vitro assays to verify the molecular docking results would be the next logical step, adding a level of validation to the in silico findings. Consequently, conducting in vivo studies, such as clinical trials, is crucial to determine the effectiveness and safety of these compounds on human skin. This would serve as the definitive assessment of their potential as anti‐ageing agents [56]. In order to enhance the accuracy of molecular docking predictions, it would be beneficial to integrate molecular dynamics simulations, which can provide valuable information about the dynamic behaviour of protein–ligand interactions over time. Enhancing the robustness of the predictions could be achieved by incorporating variables such as solvent effects, pH variations, and cofactor presence into the computational models [57].

## CONCLUSION

This study into the impact of 174 compounds known to be in extracts of a wide variety of agricultural crop and food production residues on skin protein targets associated with ageing represents a novel contribution to the field. The integration of modern drug discovery methods offers new approaches to functional product development. While many studies have focused on individual or small groups of targets, this expansive analysis offers a comparative and statistical perspective that is relatively unexplored. It is important to acknowledge the inherent limitations of computational research, such as the potential for discrepancies between theoretical predictions and empirical outcomes, as well as the lack of comprehensive information on the content of the extract, including quantitative information. Nevertheless, this study lays the groundwork for future research that integrates computational predictions with experimental validation, advancing the development of sustainable and scientifically substantiated anti‐ageing skincare formulations.

## FUNDING INFORMATION

This work was funded by the Provincial Secretariat for Higher Education and Scientific Research, Autonomous Province of Vojvodina, Republic of Serbia: 142‐451‐3008/2023‐1.

## CONFLICT OF INTEREST STATEMENT

The authors declare no conflicts of interest.

## Supporting information


Data S1.

Data S2.
