# Supplementary material for: Mining bioactive components in agricultural crop and food production residue for sustainable solutions: In silico screening for skin anti‐ageing properties
Source: Int J Cosmet Sci. 2025 Apr 15;47(5):793–806. doi: 10.1111/ics.13059 (PMC12489880; doi:10.1111/ics.13059)
Supplement: Supplementary file 1 — Data S1. [file ICS-47-793-s001.docx]

Mining Bioactive Components in Agricultural Crop Residues for Sustainable Solutions: *In Silico* Screening for Skin Anti-Aging Properties

**Table S1.** Centroid coordinates of receptor proteins.

| **Receptor** | **Coordinates (Å)** | | |
| --- | --- | --- | --- |
|  | **X** | **Y** | **Z** |
| Collagenase (1CGL) | 38.6 | 35.5 | 0.8 |
| Collagenase (2TCL) | 72.0 | 9.0 | 11.0 |
| Elastase (1ELB) | 40.0 | 22.0 | 38.0 |
| Fibronectin(1FNF) | 17.0 | 1.8 | 2.1 |
| Hyaluronidase (2PE4) | 39.4 | -26.1 | -8.2 |
| Laminin (5XAU)  Matrix metalloproteinase-2 (8H78) | 25.6 | 77.2 | 28.0 |
|  | 27.0 | 22.0 | -11.0 |
| Sirtuin 1 (4I5I) | 43.0 | -21.0 | 20.0 |
| Sirtuin 6 (3K35) | 8.4 | 21.7 | -10.9 |
| Transforming growth factor-beta type I receptor (1VJY) | 16.0 | 68.0 | 6.0 |
| Tumour necrosis factor alpha (2AZ5) | -19.0 | 73.0 | 33.0 |
| Tyrosinase (7RK7) | -9.8 | -37.1 | -24.3 |
| Tyrosinase (AlphaFold model P14679-F1) | 17.4 | -15 | -106.5 |
|  |  |  |  |


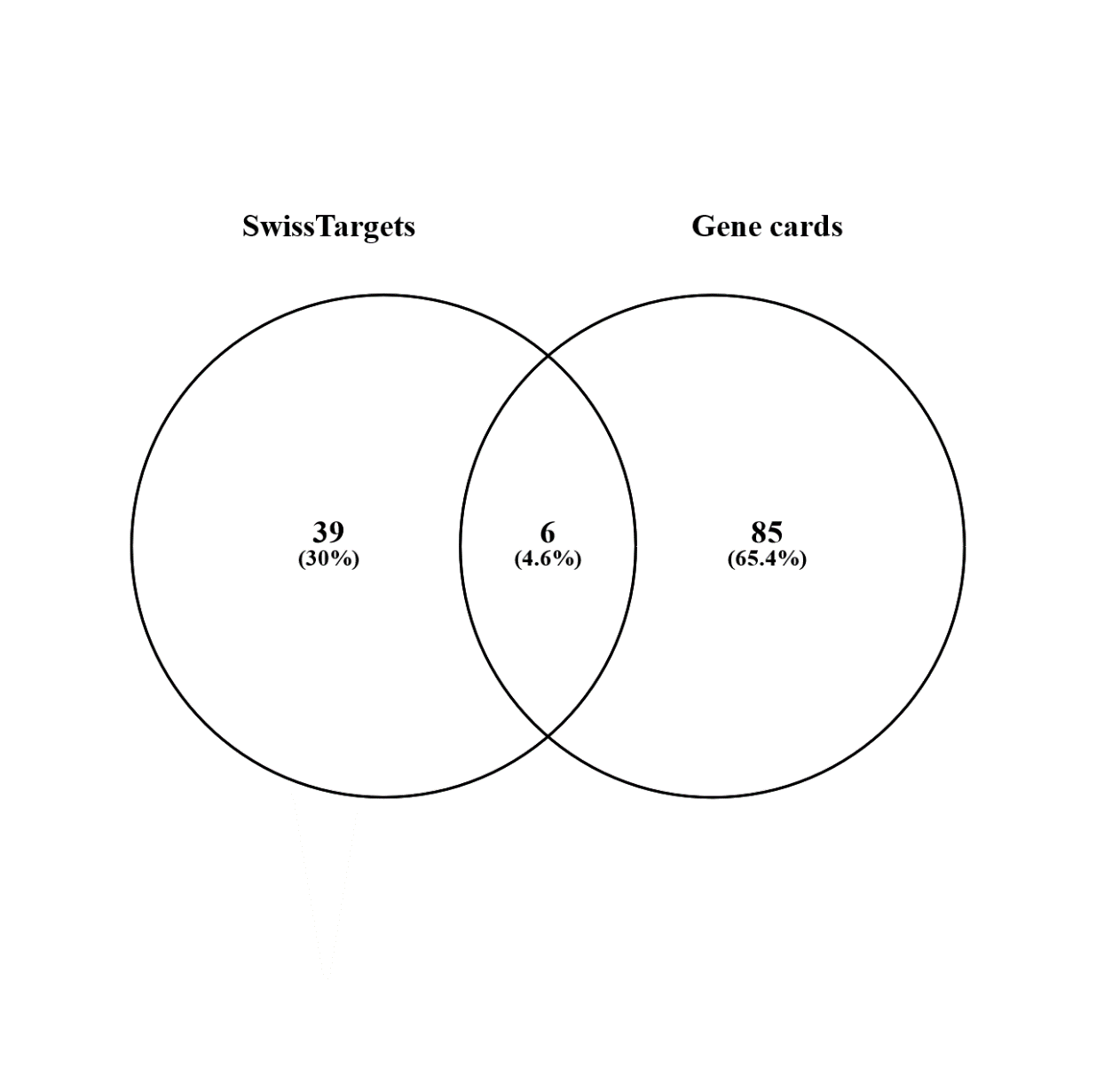


Figure S1. Overlapping target proteins found in Human Gene Database (protein encoding genes that are related to skin anti ageing) and drug targets of plum seed extract components predicted by SwissTargetPrediction webserver.


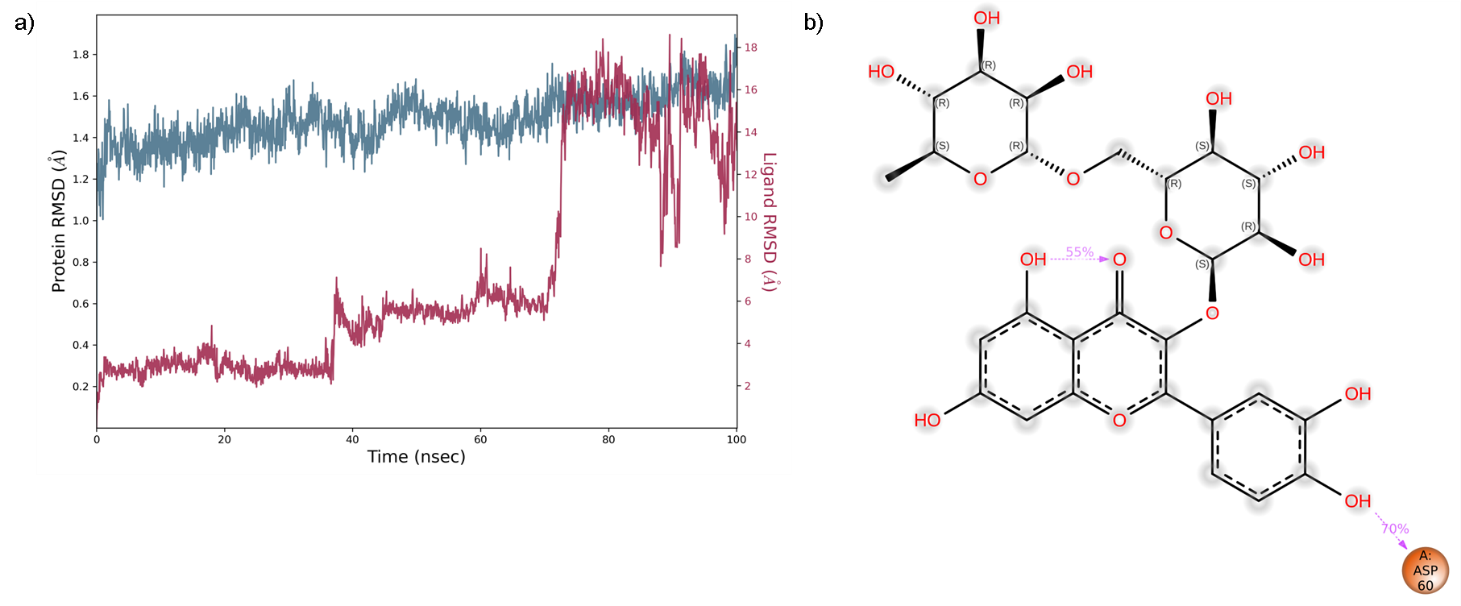


Figure S2. Selected results of the analysis of the 100 ns molecular dynamics NPT simulation at 300K conducted on neutrophile elastase (PDB ID: 8B49) in complex with rutin using Desmond software and OPLS-2005 force field. The best docking pose was used as a starting point to build a fully solvated system using explicit water and 150 mM NaCl. The RMSD plot of the 3D structure fluctuation of the protein and the ligand (a) indicates that the protein does not change the structure, however, the ligand leaves the binding site after a 70 ns production run. The protein-ligand interaction plot (b) indicates that the OH group has interacted with ASP60 for about 70 % of the simulation time, which coincides with the period that rutin has spent in the binding site. The results of this simulation indicate that molecular dynamics simulation in the presence of explicit water may not be suitable for the study of protein interactions with components of cosmetic formulations.


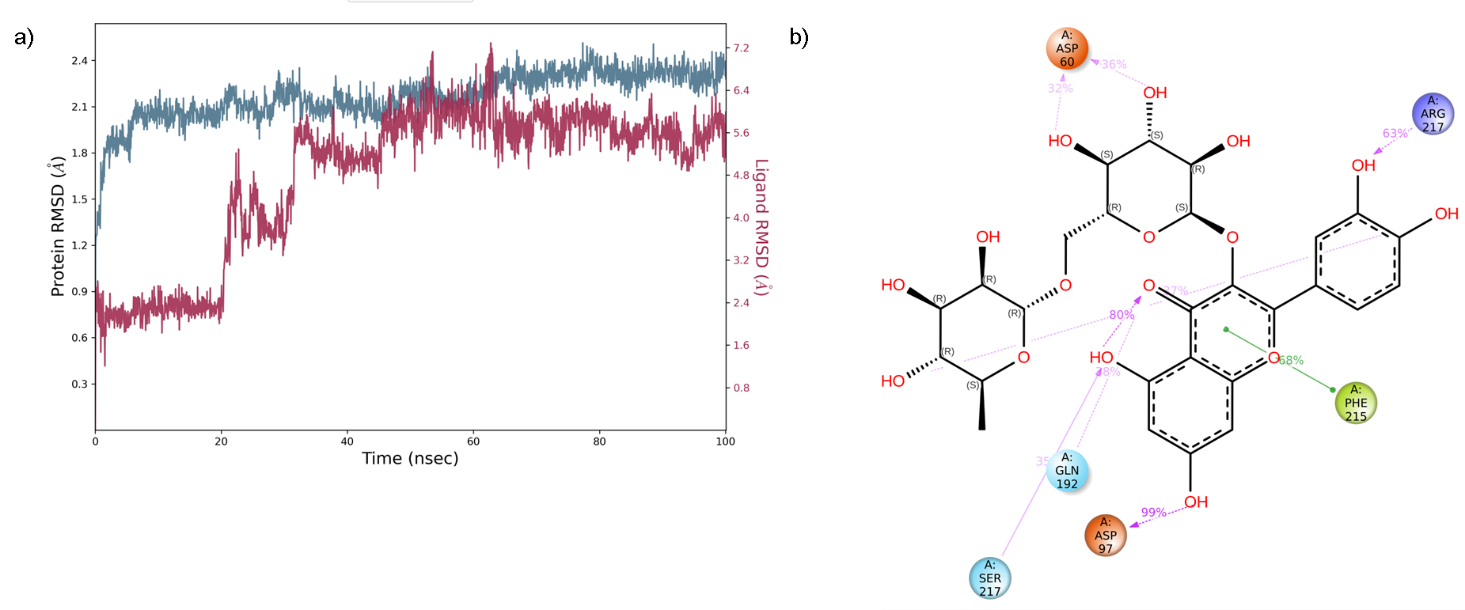


Figure S3. Selected results of the analysis of the 100 ns molecular dynamics NVT simulation at 300K conducted on neutrophile elastase (PDB ID: 8B49) in complex with rutin using Desmond software and OPLS-2005 force field. The best docking pose was used as a starting point to build a system using an implicit water model. The RMSD plot of the 3D structure fluctuation of the protein and the ligand (a) indicates that the protein does not change the structure, however, the ligand has a movement in the proximity of the binding site but it does not leave it. This is confirmed with the protein-ligand interaction plot (b) indicates that several interactions can be observed for a considerable of the simulation time, including the interaction of the OH group with ASP87 for about 99 % of the simulation time. Amino acid residues coloured in orange indicate their involvement in charged (negative) interactions, purple depicts charged (positive) interactions, cyan indicates polar interactions, and green depicts pi-pi interactions.

The results of this simulation indicate that molecular dynamics simulation using the implicit solvation model may be used as a first step in the screening of natural products to be used in cosmetic formulations.


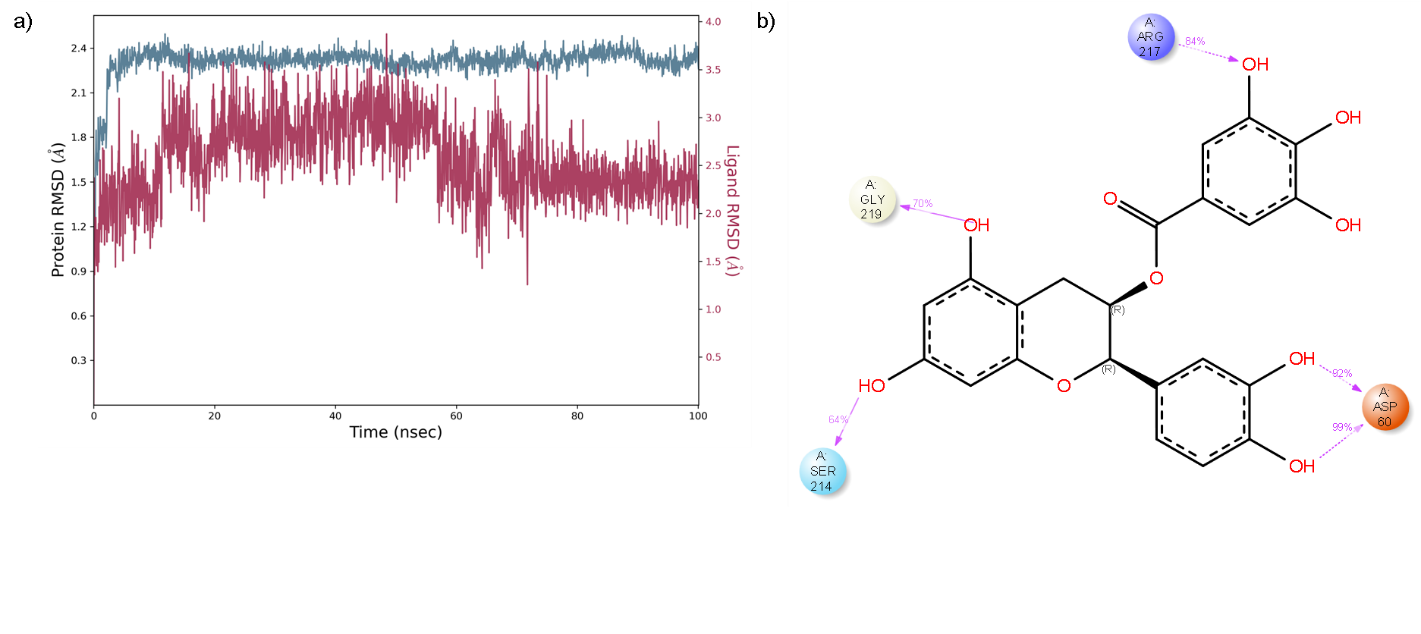


Figure S4. Selected results of the analysis of the 100 ns molecular dynamics NVT simulation at 300K conducted on neutrophile elastase (PDB ID: 8B49) in complex with epicatechin using Desmond software and OPLS-2005 force field. The best docking pose was used as a starting point to build a system using an implicit water model. The RMSD plot of the 3D structure fluctuation of the protein and the ligand (a) indicates that the protein does not change the structure, however, the ligand has a movement in the proximity of the binding site but it does not leave it. This is confirmed by the protein-ligand interaction plot (b) indicates that several interactions can be observed for a considerable of the simulation time, including the interaction of the OH group with ASP60 for about 99 % of the simulation time. Amino acid residues coloured in orange indicate their involvement in charged (negative) interactions, purple depicts charged (positive) interactions, cyan indicates polar interactions and pale yellow depicts glycine interactions.

The results of this simulation further confirm that molecular dynamics simulation using the implicit solvation model may be used as a first step in the screening of natural products to be used in cosmetic formulations.
